# Higher-contrast images are better remembered during naturalistic encoding

**DOI:** 10.1038/s41598-024-63953-5

**Published:** 2024-06-11

**Authors:** Limor Brook, Olga Kreichman, Shaimaa Masarwa, Sharon Gilaie-Dotan

**Affiliations:** 1 School of Optometry and Vision Science, Faculty of Life Science, Bar Ilan University, Ramat Gan, Israel; 2 The Gonda Multidisciplinary Brain Research Center, Bar Ilan University, Ramat Gan, Israel; 3 UCL Institute of Cognitive Neuroscience, London, UK

**Keywords:** Human behaviour, Long-term memory, Visual system

## Abstract

It is unclear whether memory for images of poorer visibility (as low contrast or small size) will be lower due to weak signals elicited in early visual processing stages, or perhaps better since their processing may entail top-down processes (as effort and attention) associated with deeper encoding. We have recently shown that during naturalistic encoding (free viewing without task-related modulations), for image sizes between 3°–24°, bigger images stimulating more visual system processing resources at early processing stages are better remembered. Similar to size, higher contrast leads to higher activity in early visual processing. Therefore, here we hypothesized that during naturalistic encoding, at critical visibility ranges, higher contrast images will lead to higher signal-to-noise ratio and better signal quality flowing downstream and will thus be better remembered. Indeed, we found that during naturalistic encoding higher contrast images were remembered better than lower contrast ones (~ 15% higher accuracy, ~ 1.58 times better) for images at 7.5–60 RMS contrast range. Although image contrast and size modulate early visual processing very differently, our results further substantiate that at poor visibility ranges, during naturalistic non-instructed visual behavior, physical image dimensions (contributing to image visibility) impact image memory.

## Introduction

Many studies have shown that visual memory is influenced by conceptual information as visual category and emotional content^1–3^. Furthermore, it has also been substantiated that deeper processing (associated with higher-level aspects of information) leads to better memory than shallower processing (associated with lower-level information)^4–6^ suggesting that visual memory is significantly influenced by higher (perceptual and conceptual) aspects of the stimulus, and much less attention has been given to the influence of lower aspects (e.g. physical) of the stimulus on memory. Most visual memory studies are often performed under task-related conditions (participants are asked to encode the stimuli either directly (e.g. memorize the images^3,7–10^) or indirectly (make non-memory-related judgements about the images^3,11^)) which could also contribute to the dominance of higher-level cognitive influences on memory. We have recently found that during naturalistic encoding (without task-related modulations) bigger images are better remembered even when they do not convey more information^12^. This was in line with our hypothesis that without task-related modulations the enhanced visual system resources recruited to process bigger images (relative to smaller ones) would result in stronger image memory.

Memory-related areas are known to receive inputs from higher-level visual areas (e.g.^13^) that receive inputs from earlier stages of visual processing (early visual cortex). Luminance contrast is known to affect early stages of visual processing such that higher contrast leads to stronger preferential responses (retina^14^, LGN^15^, V1^16–19^). While in higher-level visual areas there is a certain level of contrast invariance^20,21^, there are indications that without top-down influences of attention, high-order visual cortex activity may also be modulated by contrast similarly to V1^22^. Therefore, here we reasoned that without top-down task-related modulations, the elevated activity in early visual stages caused by higher contrast will result in higher signal-to-noise ratio (i.e. better quality) signals leading to more salient representations in higher-level visual and memory-related areas resulting in stronger consolidation of these images relative to those of lower contrast. In addition, we also assumed that the memorability of images^23,24^ which has been shown to be an image-related trait/characteristic^25^, will increase with higher contrast in a similar manner to that found for bigger image size^12^.

## Results

**Figure 1:**
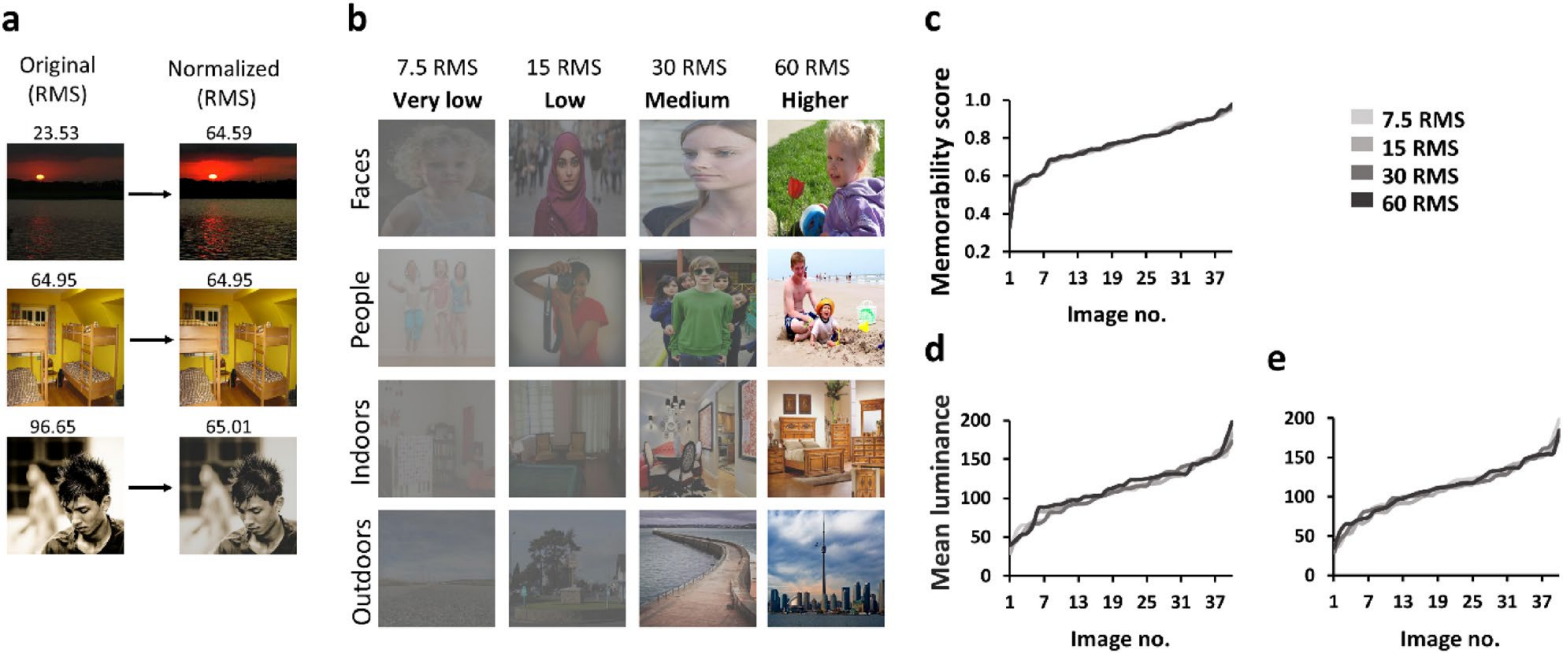
Experimental stimulus properties. (**a**) Examples of the original images from the ‘LaMem’ database (left column^2,12^) and after being converted to a uniform contrast baseline level of ~ 65 RMS (right column). Contrast values (RMS) presented above each image. (**b**) Examples of exposure phase experimental stimuli according to contrast level (7.5, 15, 30, 60 RMS, columns) and visual category (faces, people, indoors, outdoors, rows). (**c**–**e**) Images of each contrast condition (during exposure phase; shades of gray represent contrast levels from light gray-7.5 RMS to darker gray-60 RMS) are presented in an ascending order (x-axis) according to their (**c**) LaMem image memorability scores and (**d**,**e**) mean luminance level (y-axis) for each of the experimental versions. (**c**) Image memorability scores (from ‘LaMem’^2^) were equally distributed across the experimental contrast conditions as can be seen by the almost overlapping curves. (**d**,**e**) Image mean luminance levels were equally distributed across the experimental contrast conditions in version1 (**d**) and version2 (**e**) as can be seen in each panel with almost overlapping curves.

**Figure 2:**
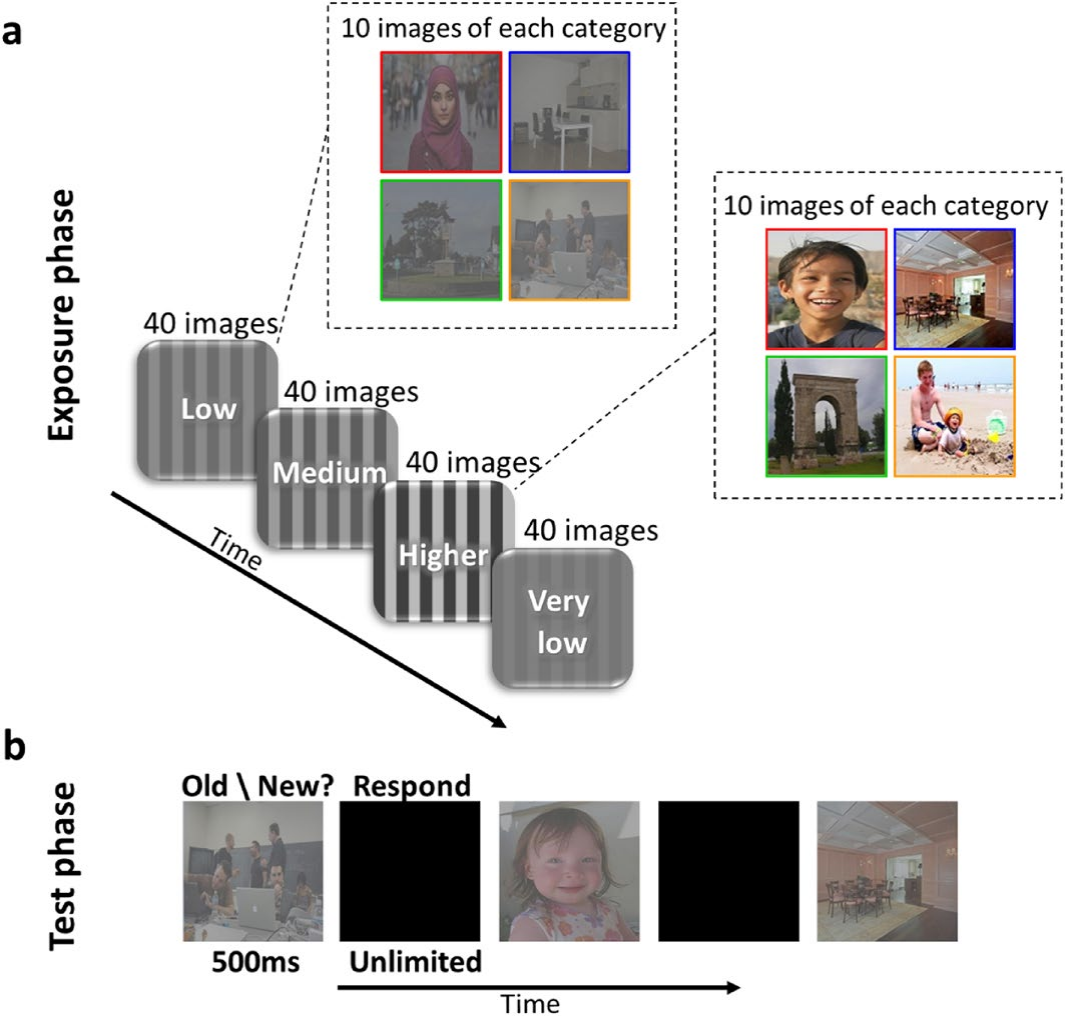
Experimental paradigm. (**a**) Exposure phase: participants (n = 47) viewed 160 images (from “LaMem” database^2^) in four blocks, each block included 40 images of a specific contrast level (7.5 RMS (very low), 15 RMS (low), 30 RMS (medium), 60 RMS (higher)) with 10 images of each visual category (faces [red], people [orange], indoors [blue], and outdoors [green]). Each image was presented once in only one of the contrast levels such that different sets of images were presented for each contrast level. Block order and within-block image order were random. Images (14.2° × 14.2°) were presented for 2 s followed by a black screen of 500 ms. Participants were instructed to freely view the images (no encoding instructions) without being informed of any memory-related task that would follow. No response was required. (**b**) Test phase: participants were given a surprise old/new recognition memory test where they viewed 320 images (160 old) in an intermediate contrast level (21 RMS, see “Methods”). Participants were required to report for each image if they recall seeing it earlier (“old”) or not (“new”). Images were presented in random order, each for 500 ms after which a black screen appeared until a response was given (with no time limit). No feedback was given.

To test these hypotheses participants (n = 47) were presented with a series of real-world images (160 14.2° × 14.2° images, 2 s/image, 500 ms ISI) of different contrast levels (7.5, 15, 30 or 60 RMS (a commonly used method to estimate color image contrast, see “Methods”); see Figs. 1 and 2). Participants were only asked to freely view the images without being informed of any memory-related task that may follow (naturalistic encoding). Across contrast levels, the images were balanced for memorability (according to their LaMem memorability scores^2,12^), mean luminance, and visual categories (faces, people, indoors, and outdoors); see “Methods” and Fig. 1. After the image exposure phase, participants were given a surprise old/new recognition memory test where they were asked to report for each image (320 (160 old) intermediate contrast (21 RMS) images, new images matched for category to the older images) if they recall seeing it earlier (“old”) or not (“new”, Figs. 1 and 2).

We first found that recognition memory performance levels (see Table 1) were similar to those found in our earlier study (image size effects on memory^12^) for both old images (average accuracy of ~ 46% across size manipulations^12^ vs. ~ 55% in the current study across contrast manipulations) and for new images (average accuracy of ~ 80% in both studies). Since there were two experimental versions (swapping the higher and lower contrast image sets in the exposure session: 7.5 with 60, 15 with 30 RMS) but there was no effect of experimental version (2-way_(version, contrast)_ ANOVA, version main effect: F(1,43) = 3.9 p = 0.055) or interaction between contrast and version (F(3,129) = 2.25 p = 0.085, Table 2), we collapsed the data across the two versions.

**Table 1:** Surprise recognition memory results at test phase according to condition.

	7.5 RMS	15 RMS	30 RMS	60 RMS	New
Accuracy (% correct)					
All categories	46.06 ± 2.7	53.56 ± 2.7	59.60 ± 2.9	61.33 ± 3.0	80.99 ± 1.9
Faces	53.78 ± 3.6	64.22 ± 3.1	66.22 ± 3.6	67.33 ± 3.1	79.18 ± 2.2
People	47.11 ± 3.4	56.15 ± 3.9	66.67 ± 3.1	67.33 ± 3.7	86.18 ± 1.7
Indoors	44.89 ± 3.9	51.11 ± 3.2	54.22 ± 3.5	58.67 ± 3.7	76.88 ± 2.4
Outdoors	38.44 ± 3.0	42.77 ± 3.5	51.31 ± 3.7	52.00 ± 4.3	81.72 ± 2.3
RT (ms)					
All categories	1092.85 ± 38.8	1145.86 ± 51.0	1119.53 ± 46.6	1113.36 ± 39.0	1132.83 ± 41.9
Faces	1072.46 ± 44.8	1109.10 ± 45.5	1074.62 ± 44.8	1057.48 ± 41.3	1143.08 ± 45.2
People	1093.18 ± 40.8	1089.35 ± 46.1	1082.36 ± 48.2	1104.85 ± 41.9	1115.12 ± 42.0
Indoors	1071.08 ± 48.5	1115.95 ± 50.2	1130.32 ± 75.3	1114.00 ± 42.6	1142.44 ± 42.4
Outdoors	1134.68 ± 51.0	1269.06 ± 132.4	1190.82 ± 80.6	1177.12 ± 70.3	1130.70 ± 45.5

Mean ± SE for accuracy (% correct) and reaction time (RT, in ms). Accuracy and RT reported for all visual categories together and below for each category separately. RT reported is relative to trial onset.

**Table 2:** Statistical analysis on accuracy.

Accuracy	Factors	Main effect	Interaction	Post-hoc (Bonferroni/Dunn)
Version X Contrast	Version Version 1, Version 2	F(1,43) = 3.9, p = 0.055	F(3,129) = 2.25, p = 0.085	
	Contrast (RMS)	F(3,129) = 25.2, **p < 10**^**−12**^		
	7.5, 15			**p = 0.003**
	15, 30			**p = 0.034**
	30, 60			p = 1
Contrast X Category	Contrast	F(3,132) = 24.6, **p < 10**^**−11**^	F(6.77,297.77) = 1.23, p = 0.28	
	7.5, 15			**p = 0.00009**
	15, 30			**p = 0.002**
	30, 60			p = 1
	Category	F(3,132) = 22.3, **p < 10**^**−11**^		
	Faces, People			p = 0.11
	People, Indoors			**p = 0.0004**
	Indoors, Outdoors			**p = 0.003**

Statistical analyses were performed using a 2-way mixed ANOVA with version_(between-subjects)_ and contrast_(within-subjects)_, and a 2-way repeated-measures ANOVA with contrast and visual category on accuracy. These were followed by Bonferroni/Dunn post-hoc tests. Values in bold represent significant results.

**Figure 3:**
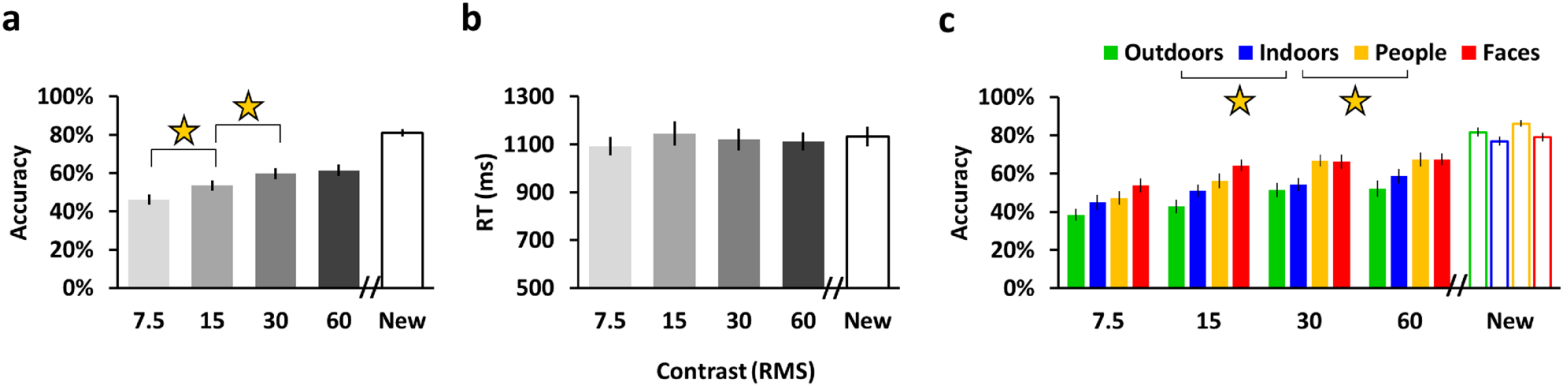
Image contrast affects image memory during naturalistic encoding (n = 45). (**a**) Test phase (surprise old/new recognition memory task) accuracy (% correct, y-axis) by condition (x-axis). A significant effect of image contrast on memory was found (p < 10^−10^) with memory increasing for image contrast between 7.5 and 30 RMS. (**b**) Test phase RT (in ms, y-axis) by condition (x-axis). No significant effect of image contrast on RT was found. (**c**) As in (**a**) and according to category. A significant effect of visual category was found (p < 10^−10^), with faces and people best remembered and outdoors the least. Error bars represent SEM. Asterisks denote significant between-condition differences. See “Results” for more details.

In line with our hypothesis we found that contrast had a significant effect on memory such that the memory for higher contrast images was better than that for lower contrast images (15.28% ± 1.80% (SEM, n = 45) higher accuracy or 1.58 ± 0.18 (SEM) times better for 60 RMS images vs 7.5 RMS images; 2-way_(contrast, category)_ ANOVA contrast main effect: F(3,132) = 24.6, p < 10^−11^ (post hoc 7.5 RMS < 15 RMS < 30 RMS; all p’s ≤ 0.002); see Table 2 and Fig. 3). The mean effect size we found (15.28%) between the high (60 RMS) and low (7.5 RMS) contrast images was comparable in magnitude (15.28% vs 15%) and much more significant than that set in the power analysis (p < 10^−9^ (60 RMS vs 7.5 RMS paired *t*-test) vs. α = 0.005 (see “Methods”)). We also replicated our earlier finding that faces were best remembered and outdoor scenes the least^12^ (visual category effect: F(3,132) = 22.3, p < 10^−11^, Fig. 3c), which is also in line with earlier findings^26,27^. In addition, there was no significant effect of contrast on RT (1-way repeated measures ANOVA: F(2.08, 91.44) = 0.82, p = 0.44, Fig. 3b).

In addition, we found that perception is unlikely to fully explain these findings of contrast affecting image memory since perceptual measures were almost at ceiling for all contrast levels as revealed by a different experiment with a different set of participants (n = 17) on these same images. Importantly, in that perceptual experiment no effect of contrast was found when participants had to report for each contrast-modulated image (same images as in our main memory experiment) whether there was a person in the image and whether the image was taken indoors or outdoors. More details and data are available at subdirectory “SuppMat_Effect of contrast on perception” at https://osf.io/9knc6/.

### Image memorability

**Figure 4:**
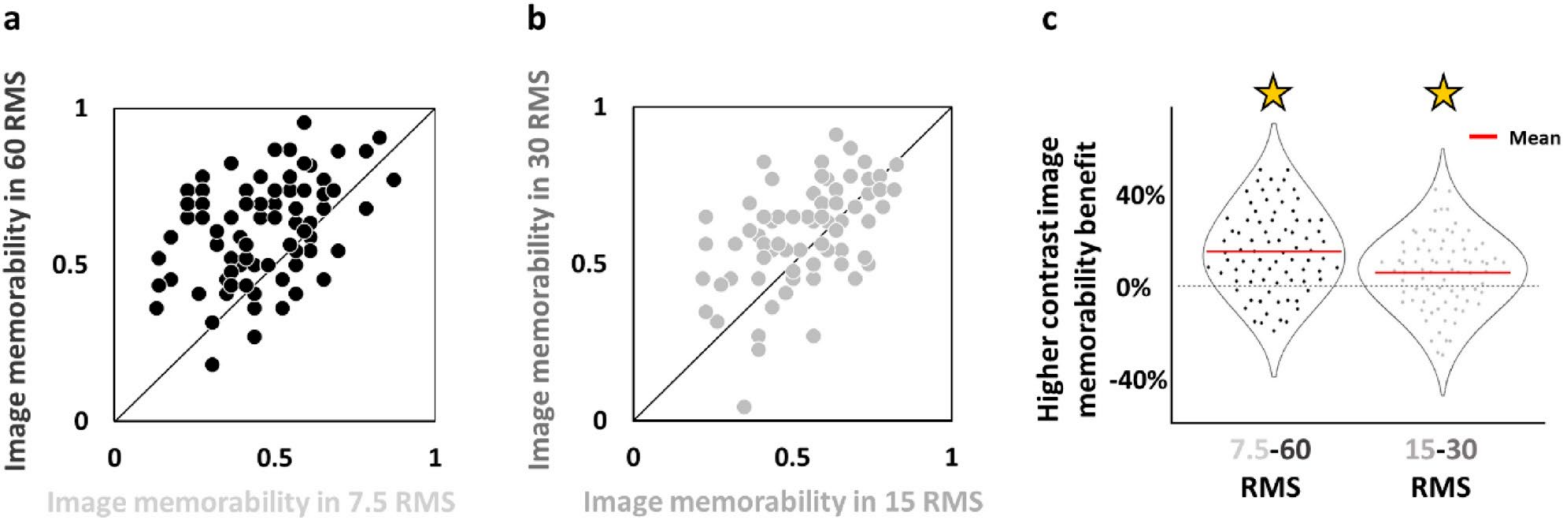
Image memorability by image contrast reveals per-image higher memorability when presented in higher contrast. (**a**) For each image (n = 80 images, each represented by a dot) its average correct recognition across participants (i.e. memorability) when presented in higher contrast (60 RMS, on the y axis) relative to its average correct recognition across participants (i.e. memorability) when presented in very low contrast (7.5 RMS, on the x-axis). Note that each image was presented in one version in higher contrast and in the other in lower contrast since the image sets swapped contrast levels between versions. Most images fall above the diagonal, indicating that they were better remembered (higher memorability) when presented in higher contrast. (**b**) The same analysis as in (**a**) for images of the two other contrast levels (30 and 15 RMS) presented. For each image (n = 80 images), its average correct recognition across participants (memorability) when it was presented in medium contrast level (30 RMS, on the y-axis) is plotted relative to when it was presented in low contrast (15 RMS, on the x-axis). Here again, most images fall above the diagonal. (**c**) Significant image contrast memorability benefit. Images were significantly more memorable when presented in higher contrast levels relative to when presented in lower contrast (60 vs 7.5 RMS: p < 10^−10^ and 30 vs 15 RMS: p < 0.001, paired *t*-tests). Data on y-axis presents the per-image difference in memorability for higher vs lower contrast presentations. Left (memorability (60 RMS)-memorability (7.5 RMS)) matches data from (**a**), right (memorability (30 RMS)-memorability (15 RMS)) matches data from (**b**). For each plot, the mean is indicated in red.

Since we found in our previous study^12^ that image size affects image memorability such that an image viewed as larger (e.g. 24° × 24°) is more likely to be remembered (higher memorability) than when it is presented as smaller (e.g. 3° × 3°), here we assumed that image contrast would also contribute to image memorability. Since we ran 2 versions of the experiment (version1: n = 24, version2: n = 23) where images presented in higher contrast in one version were presented in lower contrast in the other version and vice versa, we were able to obtain for each image its memorability when it was presented in higher contrast (60 or 30 RMS) and compare it to its memorability when it was presented in lower contrast (7.5 or 15 RMS, respectively). In line with our hypothesis that contrast would affect memorability we found that contrast level had significant effect on image memorability with ~ 15% higher memorability found for higher contrast relative to lowest contrast (paired 1-tailed *t*-tests: 60 vs 7.5 RMS: p < 10^−10^) and ~ 6% higher memorability for medium relative to the low contrast (paired 1-tailed *t*-tests: 30 vs 15 RMS: p < 0.001, see Fig. 4).

## Discussion

While it is commonly assumed that physical image information does not significantly affect image memory, here we assumed that image visibility plays an important role in image memory when no task-related modulations are involved. Following our recent results showing that image size affects memory during naturalistic encoding, here we tested whether image luminance contrast that significantly affects image visibility, contributes to image memory. We found that in lower visibility ranges image memory is significantly affected by contrast (up to 30 RMS).

Higher contrast images appear easier to perceive than lower contrast images and therefore one logical assumption stemming from this is that higher contrast images will be better remembered. On the other hand, lower contrast images are more difficult to perceive and therefore are likely to recruit during the encoding phase more resources (as effort or attention related). These additional resources may contribute to deeper processing of these lower contrast images and thus may lead to better memory for these images^4–6^. It is therefore not straightforward whether higher contrast images will be better remembered or not. One study tested the perceptual fluency hypothesis that suggests that items easier to perceive create an illusion that they will be better remembered^28^. They found that people indeed estimated their memory to be higher for intact images than for generate images (as the perceptual fluency hypothesis suggests) but their actual memory recall for the generate images was at least as high as and sometimes even higher than that of intact images. Another study found that images of poorer visibility (blurry, pixelated, etc.) were remembered as much more visible than they actually were at the encoding stage. The authors termed this phenomenon “vividness extension”^29^ and it is unclear whether vividness extension applies to image contrast that was not tested in that study. There are also additional indications that memory sometimes distorts and may not genuinely reflect the physical stimulus properties that were present at encoding (e.g. boundary extension and boundary contraction^30,31^). Since it was unclear whether memory for higher contrast images would be higher than for lower contrast images, here we tested this and as in our previous study, this was performed without task-based modulations (naturalistic encoding) in an effort to closely mimic naturalistic daily visual behavior. Our finding that contrast levels influence memory (for the range of up to 30 RMS) during such naturalistic encoding conditions may not generalize to other non-naturalistic encoding conditions. In addition, we did not directly measure activation levels and patterns within and beyond the visual system and therefore we cannot determine if our assumptions, that this effect is due to higher activations in early visual stages, is indeed the cause for the heightened memory levels for higher contrast images that we found.

Image size and image contrast modulations affect visual processing in a very different manner. While image size modulation influences visual processing by recruiting more resources (larger areas) across early to mid-processing stages (e.g. bigger portions of the retina, LGN and retinotopic cortex are activated^20,32^), image contrast modulation are considered to activate more strongly the already activated network^14,15,19^. Despite these differences in early visual processing, we found that downstream memory processes are influenced in a comparable manner (image contrast, like image size, modulates visual memory). We hypothesize that both bigger size and higher contrast images result in higher signal-to-noise-ratio information entering downstream high-order visual areas thus likely influencing memory processes in a corresponding manner.

While most visual processes involve both bottom-up and top-down components, it is often assumed that higher-level visual processes show more invariance to low-level (bottom-up) stimulus dimensions (e.g. change of contrast or size^33^). However, such cue-invariances in high-level visual cortex were typically found during task-based paradigms (e.g.^20,21,33,34^). We assume that certain levels of contrast and size invariance found in high-level visual areas in these previous studies may reflect enhanced attention to task-relevant stimulus features over physical stimulus properties. This idea is in line with a recent study^35^ investigating the effects of top-down tasks on high-level visual processing finding that activations across the cortex and specifically within ventral temporal cortex were modulated by top-down task-driven attention. Furthermore, another study^22^ found that for unattended stimuli, LOC’s activity was modulated by contrast in a similar manner to that of V1. These results may suggest that high-level visual areas may be less contrast or size invariant during naturalistic visual behavior than previously assumed.

Image memorability has been shown to be an image-specific trait^23–25,36^. Here we found in an image-based analysis that image memorability was affected by contrast with higher contrast leading to higher memorability (Fig. 4). These results are in line with our earlier findings that the physical dimension of image size also affects image memorability with bigger size leading to higher image memorability^12^. While image memorability has not been attributed to the image’s physical properties, we assume that our results highlight the modulation that image memorability undergoes in visibility ranges that are lower than those investigated earlier (i.e. 7.5–60 RMS, 3°–24°; based on our experimental findings most likely the most sensitive ranges are 7.5–30 RMS and 3°–12°) and are not likely to extend substantially beyond these ranges.

While the overall memory performance we found here following contrast modulations is similar to that found for image size modulations^12^, it is substantially lower than that found in earlier encoding-based studies^3,7,37^. The lack of encoding task as well as the possibly low level of conceptual distinctiveness^38^ of our stimuli (limited by the 4 conceptual categories our stimuli came from), which may have led to within-category representational interference^39^, may have both contributed to these effects. Furthermore, since our study was performed on 4 categories (faces, people, indoors, outdoors) we cannot determine whether our results will generalize to additional visual categories and to what extent. It is also important to note that here we parametrically modulated luminance contrast and not color contrast^40,41^. Nevertheless, our luminance contrast modulation has also influenced the stimuli’s colorfulness and visibility in general and thus we cannot rule out the possibility that colorfulness by itself may influence image memorability^10^. Our study did not parametrically modulate stimulus luminance (note that luminance levels were matched between the different conditions as can be seen in Fig. 1d,e) and did not investigate potential effects of mean luminance levels on memory. Furthermore, since our results were obtained during naturalistic encoding they may not generalize to experiments with top-down modulations-based task that typically involve predetermined experimental direct or indirect encoding tasks. These findings were obtained with young adults aged 18–35 years, an age range where vision is already fully developed but aging-related effects are not typically present. Since contrast sensitivity changes across the life span (e.g.^42^), it is unclear whether these results will generalize to additional age groups. Our results may also reflect multiple (and possibly additive) effects that are driven by image contrast changes such as changes in image saliency, spatial frequencies, or eye movement patterns. Importantly, our results that image contrast affects memory imply that image contrast is one of multiple factors contributing to image memory together with additional lower- and higher-level factors.

Earlier studies show that memory is influenced by top-down modulations^4–6^. Our findings highlight the contribution of bottom-up (low-level) processes to visual memory and together with earlier studies suggest that during naturalistic visual behavior there is likely an interplay between bottom-up and top-down (higher-level) processes that determines their combined influence on visual memory. Furthermore, we hypothesize that both bottom-up and top-down processes are likely to be part of almost all visual processes and that the degree of their contributions is determined by the task at hand.

## Methods

### Participants

A group of 47 young adult participants took part in this study (31 women, aged 24.4 ± 4.04 (SD) years (all in the age range of 18–35 years where vision is fully developed but no aging effects are likely to be evident), 44 right handers, all with normal or corrected to normal vision (visual acuity (VA) checked before the main experiment began)). Of the 24 participants that underwent version1 (see below) and 23 that underwent version2 (see below), two participants were excluded (one from each version, both right-handed, one woman aged 24 years wearing her glasses, one man aged 34 years with normal vision) since their accuracy performance in the new condition (details below) was more than 2 standard deviations below the group mean. VA was measured in all but 4 participants (for 3 of them the VA measuring chart was not available on the day they were tested, another could not return to complete the VA measurement). The demographic information we collected included age, gender, handedness, and vision related measures (as VA and whether participants did or did not need correction with contacts or glasses, see full details at https://osf.io/9knc6). While we do not have further demographic information, many of participants were university students, many living in urban areas, and all were living in a western country (therefore we can assume that the vast majority had high school education). Sample size was estimated based on six experiments from our previous study^12^ with a similar paradigm (all were different participants than in the current study) in which we tested the effect of picture size (small and large) on memory recognition accuracy (%) during naturalistic encoding. Specifically, we tested the difference in accuracy between large and small picture conditions for each group of 16–26 individuals (i.e. paired samples). Based on the standard deviations of the differences and expecting in this study a mean difference of at least 15% in accuracy between the highest and lowest contrast conditions (60 RMS vs 7.5 RMS with α = 0.005, β = 0.9), the largest sample size required was estimated to be n = 24. Therefore, here we ran 2 experimental versions (see Procedure), each with a sample size of ~ 24 participants. All participants signed a written informed consent form before their participation. The study was approved by the Bar Ilan University Multidisciplinary Research Unit ethics committee.

### General procedure

This experiment was run on a Windows PC equipped with an Eizo FG2421 24″ HD LCD 100 Hz display with 1920 × 1080 pixel resolution in a dark room with viewing distance of 60 cm using an in-house platform for psychophysical experiments [PSY (Yoram S. Bonneh)^12,43,44^]. The experiment took ~ 30 min. ANOVA and post-hoc statistical analyses were performed using R studio (version 2021.9.1.372)^45^, Bonferroni/Dunn post-hoc analyses were used.

The experiment included two consecutive parts (Fig. 2). First, the exposure phase with passive viewing of real world images of different contrast levels where participants were asked to freely view and attend the images presented without being informed of any memory-related task that would follow or the contrast manipulations that the images underwent. Second, the test phase with an old/new surprise recognition memory task where participants were asked to report for each images if they recalled seeing it earlier (“old”) or not (“new”). The background color of the screen across all experiments was always black and all images subtended 14.2° × 14.2° of visual angle.

### Procedure

At the exposure phase, 160 images were presented in four blocks. Each block presented 40 images of a specific contrast (7.5, 15, 30, or 60 RMS, for more details see Stimuli section) with 10 images of each visual category (faces, people, outdoors, indoors). Block order and image order within each block were random (Figs. 1 and 2). There were two versions of the exposure session (version1: n = 24, version2: n = 23, each participant participated in only one version); image sets assigned to the 60 RMS condition in version1 were assigned to the 7.5 RMS condition in version2 and vice versa, and image sets assigned to the 30 RMS condition in version1 were assigned to the 15 RMS condition in version2 and vice versa. This was done to rule out the possibility that the results were driven by the specific image sets used for each contrast group in one of the versions. Each image was displayed for 2 s followed by a 500 ms black screen ISI. Participants were asked to freely view and attend the images, fixation or response were not required (Fig. 2).

A test phase followed the exposure phase where participants were required to perform a surprise old/new recognition memory task on 320 images: 160 “old” (previously seen in the exposure phase) and 160 “new” images (not seen in the exposure phase), old and new images were presented in random order. All images were presented at the same intermediate contrast level (21 RMS, see below). Each image appeared for 500 ms followed by a black screen until a response was provided; participants were required to report if they recalled seeing each image (old) or not (new) without time limitations; no feedback was given (Fig. 2).

### Stimuli

All images used in this study were originally taken from the “LaMem” Dataset with predefined memorability scores for each image^2^ (same 320 original images used in our earlier study^12^). All images (320 images) were first resized in MATLAB 2018b (MathWorks Inc.) by the imresize function (bicubic interpolation) to 900 × 900 pixels to avoid within- and across-condition size differences (for more details see^12^). We then modulated the contrast level of each image using in-house MATLAB-based scripts (version 2018b (MathWorks Inc.)) we developed (code provided in https://osf.io/9knc6/) in a 2-stage process. All images were first brought to a uniform contrast baseline level of 65 RMS (according to the mean contrast of all images before contrast manipulations, Fig. 1a). From this uniform contrast level, we then further manipulated the contrast level of each image according to the experimental condition it was to be presented in (four contrast level conditions in the exposure phase (7.5 RMS (very low), 15 RMS (low), 30 RMS (medium), 60 RMS (higher)), and one fixed (intermediate) contrast level in the test phase (21 RMS, see details below)).

Image contrast was calculated using the root mean square (RMS) method^46–49^ that is suited for color images according to the following equation:

$${Contrast }_{(RMS)}=\sqrt{\frac{1}{MN}\sum_{i=1}^{M}\sum_{j=1}^{N}{({I}_{ij}-\overline{I })}^{2}}$$

where M and N stand for the number of rows and columns in the image matrix respectively, *I*_*ij*_ stands for the value of the pixel in the ith row and jth column and $\overline{I }$ stands for the average value over all pixels in the image (taking into account the three color channels (red, green and blue (RGB)) together).

For each of the contrast-manipulated experimental images we estimated the average luminance level determined as the mean luminance level over all the image pixels after it was converted to grayscale (using the rgb2gray MATLAB function). In the exposure phase the 4 image sets (one for each of the 4 contrast level conditions) were well balanced for memorability scores (according the their LaMem memorability scores^2,12^), mean luminance levels, and had equal contribution of each visual category (Fig. 1). The 160 test phase new images matched the exposure phase image-category distribution (40 faces, 40 people, 40 indoor and 40 outdoor images) and intermediate contrast level was used for all images presented at test.

*Test phase intermediate contrast level* was chosen as a logarithmic contrast midway (c_mid_ = 21.21 RMS) between the lowest (7.5 RMS) and highest (60 RMS) contrast levels used in the exposure phase such that 7.5*α = c_mid_ and c_mid_*α = 60. As contrast and contrast sensitivity are often measured in log scale^50,51^, we assumed that if visual memory during naturalistic encoding would be sensitive to contrast it would follow a similar non-linear pattern.

We also estimated image memorability independently from of the memorability scores obtained from LaMem dataset which appear in Fig. 1c. Our image memorability independent estimations were determined for each old image according to its average correct recognition across the participants that saw it in that contrast level in the exposure phase. Since each image was presented at a different contrast level in each of the different versions (half of the images were presented at 7.5 RMS in one version and 60 RMS in the other version, while the other half were presented at 15 RMS in one version and 30 RMS in the other) we were able to compare for each image how contrast level affected its memorability and these analyses are presented in Fig. 4 and in the OSF repository https://osf.io/9knc6 in the imageInformation_final.xlsx file.

## Data Availability

The data that support the findings of this study are available in the Open Science Framework repository at https://osf.io/9knc6/
